# Extracellular Superoxide Dismutase Protects *Histoplasma* Yeast Cells from Host-Derived Oxidative Stress

**DOI:** 10.1371/journal.ppat.1002713

**Published:** 2012-05-17

**Authors:** Brian H. Youseff, Eric D. Holbrook, Katherine A. Smolnycki, Chad A. Rappleye

**Affiliations:** Departments of Microbiology and Microbial Infection and Immunity, Ohio State University, Columbus, Ohio, United States of America; University of Melbourne, Australia

## Abstract

In order to establish infections within the mammalian host, pathogens must protect themselves against toxic reactive oxygen species produced by phagocytes of the immune system. The fungal pathogen *Histoplasma capsulatum* infects both neutrophils and macrophages but the mechanisms enabling *Histoplasma* yeasts to survive in these phagocytes have not been fully elucidated. We show that *Histoplasma* yeasts produce a superoxide dismutase (Sod3) and direct it to the extracellular environment via N-terminal and C-terminal signals which promote its secretion and association with the yeast cell surface. This localization permits Sod3 to protect yeasts specifically from exogenous superoxide whereas amelioration of endogenous reactive oxygen depends on intracellular dismutases such as Sod1. While infection of resting macrophages by *Histoplasma* does not stimulate the phagocyte oxidative burst, interaction with polymorphonuclear leukocytes (PMNs) and cytokine-activated macrophages triggers production of reactive oxygen species (ROS). *Histoplasma* yeasts producing Sod3 survive co-incubation with these phagocytes but yeasts lacking Sod3 are rapidly eliminated through oxidative killing similar to the effect of phagocytes on *Candida albicans* yeasts. The protection provided by Sod3 against host-derived ROS extends in vivo. Without Sod3, *Histoplasma* yeasts are attenuated in their ability to establish respiratory infections and are rapidly cleared with the onset of adaptive immunity. The virulence of Sod3-deficient yeasts is restored in murine hosts unable to produce superoxide due to loss of the NADPH-oxidase function. These results demonstrate that phagocyte-produced ROS contributes to the immune response to *Histoplasma* and that Sod3 facilitates *Histoplasma* pathogenesis by detoxifying host-derived reactive oxygen thereby enabling *Histoplasma* survival.

## Author Summary

*Histoplasma capsulatum* is a fungal pathogen that is endemic to the Mississippi and Ohio River valleys. An estimated 200,000 infections occur annually in the United States. *Histoplasma* is adept at surviving within both neutrophils and macrophages, which normally kill fungal cells by producing reactive oxygen molecules that are toxic to microbes. In this study, we demonstrate the role of a superoxide dismutase enzyme (Sod3) produced by *Histoplasma* cells and we show that it enables *Histoplasma* to survive these reactive oxidative molecules produced by the host. We show that *Histoplasma* directs the Sod3 protein to the surface of yeast cells and into the extracellular environment, positioning it to destroy extracellular superoxide produced by neutrophils and macrophages. Our results highlight the importance of reactive oxygen produced by immune cells and define the mechanism by which *Histoplasma* survives these immune defenses and establishes infections in its host.

## Introduction

Highly reactive oxygen metabolites are one of the primary effector mechanisms used by the host immune system to control or clear microbial infections. Initial host defenses against fungal invaders rely on responses of innate immune cells, particularly neutrophils (polymorphonuclear leukocytes; PMNs) and macrophages. These phagocytes produce reactive oxygen molecules through activation of the NADPH-oxidase complex that generates superoxide. Superoxide and the other reactive molecules derived from it, including peroxide and hydroxyl radicals, are collectively termed reactive oxygen species (ROS). These species can directly damage macromolecules on or in the microbe leading to death of the microbe [1]. Cytokine activation of macrophages during the adaptive immune response enhances the ability of macrophages to produce ROS and this correlates with increased ability to restrict or kill microbes [2], [3]. Since ROS molecules are highly toxic to microbes, effective pathogens must avoid or neutralize phagocyte-derived ROS in order to survive within the host. This is particularly necessary for intracellular pathogens.

The yeast form of *Histoplasma capsulatum* is an intracellular pathogen that successfully infects and parasitizes phagocytes. The fungus is widespread in the Midwestern United States and throughout Latin America. It is estimated that 200,000 infections occur annually in the United States through inhalation of infectious particles [4]. Macrophages efficiently ingest pathogenic *Histoplasma* yeast cells, but are unable to kill the yeasts [5]–[7]. By itself, the innate immune system is insufficient to clear the infection. With activation of the adaptive immune response and the corresponding enhancement of phagocyte antifungal defenses, most immunocompetent individuals can restrict *Histoplasma* proliferation [8], [9].

The mechanisms *Histoplasma* employs to avoid clearance by innate immune cells are essential to its virulence. *Histoplasma* survival in macrophages may result, in part, by the lack of an oxidative burst from these phagocytes [5], [10]–[12]. Activation of macrophages by cytokines primes their production of ROS in response to *Histoplasma* [5], [12], [13]. PMNs also participate in the initial response to respiratory *Histoplasma* infection [14], [15] and the interaction of *Histoplasma* with these phagocytes triggers a respiratory burst [16]–[19]. Nevertheless, *Histoplasma* yeast cells are not killed despite ample ROS production adequate to kill other fungi such as *Candida* [17]–[20]. How *Histoplasma* yeasts endure this oxidative challenge and the factors enabling *Histoplasma* survival in the face of phagocyte-derived ROS are unknown.

To identify new factors facilitating *Histoplasma* pathogenesis, we recently examined the extracellular proteome of pathogenic-phase *Histoplasma* cells [21]. From this, we identified three proteins potentially involved in defending *Histoplasma* from ROS. One protein is a predicted superoxide dismutase (Sod3) whose expression is greatly enriched in pathogenic phase yeast cells suggesting its importance to virulence. This extracellular protein is distinct from other *Histoplasma* superoxide dismutases that have a higher degree of similarity to cytoplasmic enzymes [21], suggesting Sod3 functions in combating extracellular superoxide, such as that which would be produced by phagocytes.

In this paper, we define the contribution of Sod3 to *Histoplasma* pathogenesis and the role of host-derived reactive oxygen defenses. To functionally test the role of Sod3, we created a *Histoplasma* strain lacking the *SOD3* gene. Our results demonstrate that Sod3 is the major source of extracellular superoxide dismutase activity and that Sod3 prevents superoxide-dependent killing of *Histoplasma* yeast cells. In addition, we show that host control of *Histoplasma* infection requires ROS production. These results identify Sod3 as an essential virulence factor of *Histoplasma* and provide a mechanistic explanation as to how *Histoplasma* survives ROS generated by host phagocytes during infection.

## Results

### The *Histoplasma SOD3* gene encodes an extracellular superoxide dismutase

Proteomic analysis of the extracellular proteins produced by pathogenic-phase *Histoplasma* cells identified a putative superoxide dismutase [21]. The gene encoding this protein was designated *SOD3* to distinguish it from the intracellular Sod proteins, Sod1, Sod2, and Sod4. The *SOD3* locus contains a 771 base pair gene comprised of two exons (GI: EEH08361.1). Previous work demonstrated that the *SOD3* gene is preferentially expressed by *Histoplasma* pathogenic yeast cells compared to expression by non-pathogenic mycelia. Furthermore, *SOD3* is expressed during in vivo infection consistent with a role in virulence [21]. In vitro, the Sod3 protein is produced by yeasts during growth in rich (HMM) and minimal (3M) media at 37°C with 5% CO_2_/95% air (data not shown).

**Figure 1:**
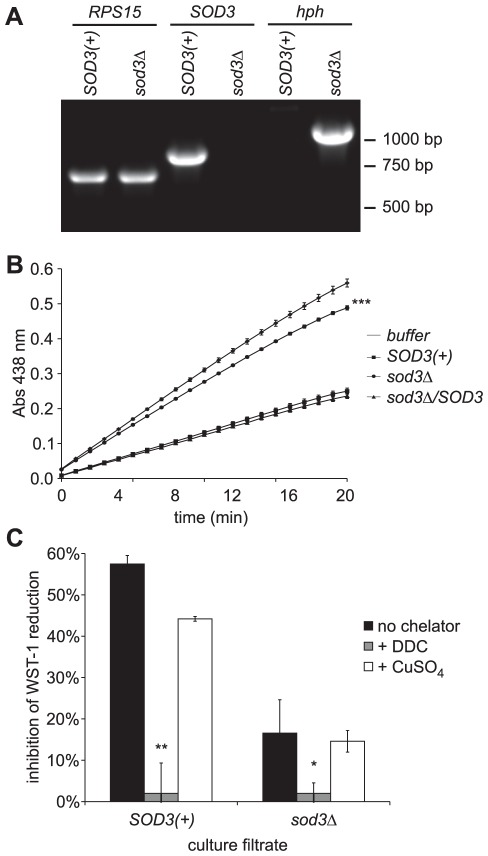
*Histoplasma* Sod3 encodes an extracellular Cu^++^-dependent superoxide dismutase. (**A**) PCR validation of deletion of the *SOD3* gene. Genomic DNA from the *SOD3(+)* parental strain (WU8) and the *sod3Δ* strain (OSU13) were tested by PCR for the ribosomal subunit gene *RPS15*, the wild-type *SOD3* gene, and the mutant allele marked with the hygromycin resistance gene (*hph*). (**B**) Superoxide dismutase activity in culture filtrates harvested from *SOD3(+)* (OSU45), *sod3Δ* (OSU15), and the *sod3Δ/SOD3* complemented (OSU49) strains. Detection of superoxide was determined through superoxide-dependent reduction of the WST-1 tetrazolium dye after generation of superoxide using hypoxanthine and xanthine oxidase. Reduction of WST-1 was monitored by absorbance at 438 nm. Buffer or culture filtrates contained 5 µg ovalbumin or total culture filtrate protein, respectively. Asterisks represent significant difference (*** p<0.001) in the inhibition of WST-1 reduction between *SOD3(+)* and *sod3Δ* culture filtrates. Data shown is representative of three independent experiments, each performed with triplicate samples. (**C**) Sod3 activity following Cu^++^ depletion. Culture filtrates containing 5 µg total protein from *SOD3(+)* (OSU45) and *sod3Δ* (OSU15) strains were tested for their ability to inhibit WST-1 reduction by superoxide before (no chelator), after Cu^++^ depletion (+DDC), and after subsequent repletion with 50 mM Cu^++^ (+CuSO_4_). Values represent relative inhibition of WST-1 reduction by culture filtrate samples (n = 3) compared to buffer controls treated in parallel. Asterisks represent significant differences from *SOD3(+)* culture filtrates (* p<0.05, ** p<0.01).

To validate the predicted *SOD3* gene and to enable functional analyses of Sod3, we created a mutant strain lacking the *SOD3* gene. A deletion allele was created by replacement of the *SOD3* coding region with a constitutively-expressed hygromycin resistance gene (*hph*). Two kilobases of DNA sequence corresponding to sequence immediately 5′ and 3′ of the *SOD3* gene flanked the *hph* cassette to serve as recombination substrates. The chromosomal locus was replaced with the deletion allele by allelic exchange and the rare recombinants were isolated by successive positive and negative selections [22]. We confirmed deletion of the *SOD3* gene by PCR analysis using primers specific for either the wild-type or the deletion allele (Figure 1A). Whereas *SOD3* coding region-specific primers detect a 770 base pair product from the parental strain (*SOD3(+)*), no *SOD3* coding region is amplified from the *SOD3*-deletion (*sod3*Δ) strain. In contrast, a PCR product corresponding to the *hph*-marker was only amplified from the *sod3*Δ strain as expected for replacement of the wild-type locus with the deletion allele. Amplification of the *RPS15* gene indicates that the genomic DNA used was competent for PCR amplification. The *sod3Δ* strain has identical colony morphology and grows at an equal rate to the *SOD3(+)* strain in culture, in both HMM and 3M media (data not shown).

Culture filtrate from the *sod3*Δ strain demonstrates that *SOD3* encodes the major extracellular superoxide dismutase activity produced by *Histoplasma* yeasts. To measure superoxide dismutase activity, we used an indirect assay of superoxide anion through superoxide-dependent reduction of the water soluble tetrazolium salt WST-1 [23]. Xanthine oxidase and hypoxanthine were used to generate the superoxide in this in vitro system. Five micrograms of total culture filtrate protein from *SOD3(+)* and *sod3*Δ strains were added to the superoxide generating/WST-1 system (Figure 1B). Culture filtrate from the *SOD3(+)* strain substantially decreased the rate of WST-1 reduction, indicating depletion of superoxide by the Sod3-containing culture filtrate. In contrast, inhibition of WST-1 reduction by culture filtrate from the *sod3Δ* strain is nearly identical to the buffer control, indicating very little destruction of superoxide by the *sod3*Δ culture filtrate. Expression of the predicted *SOD3* genomic DNA in the *sod3Δ* mutant (*sod3*Δ/*SOD3*) fully complements the loss of superoxide dismutase activity in *sod3Δ* culture filtrate. These data indicate that *SOD3(+) Histoplasma* yeasts produce soluble extracellular superoxide dismutase activity and that the *SOD3* gene product is responsible for the majority of this superoxide dismutase activity.

Bioinformatic analyses suggested Sod3 is more similar to Cu/Zn Sod proteins than to Fe/Mn-type enzymes and we functionally confirmed this by testing Sod3-dependent superoxide dismutation following copper depletion. Culture filtrates from Sod3-producing and Sod3-deficient strains were treated with 50 mM diethyldithiocarbamate (DDC), a copper chelator, to remove copper ions from Sod3 and the resultant culture filtrates tested for superoxide dismutase activity (Figure 1C). Without DDC treatment, culture filtrate from *SOD3(+)* yeasts inhibits WST-1 reduction by nearly 60% compared to the buffer control due to superoxide dismutase activity. However, DDC treatment effectively negates the inhibition caused by culture filtrate from the *SOD3(+)* strain, indicating loss of superoxide dismutase activity. DDC treatment of culture filtrate from the *SOD3(+)* strain is similar to untreated culture filtrate from the *sod3*Δ mutant, consistent with loss of Sod3 activity due to Cu^++^ chelation (Figure 1C). Culture filtrate from the *sod3Δ* mutant similarly lacks dismutase activity after treatment with DDC. Replenishing Cu^++^ ions to DDC-treated *SOD3(+)* culture filtrate largely restores superoxide dismutase activity (44% inhibition of WST-1 reduction compared to 57% inhibition with untreated *SOD3(+)* culture filtrate). These results demonstrate that Sod3 depends on copper for enzymatic activity and classifies it as a Cu/Zn-type superoxide dismutase.

### Sod3 is both secreted from and associated with yeast cells

**Figure 2:**
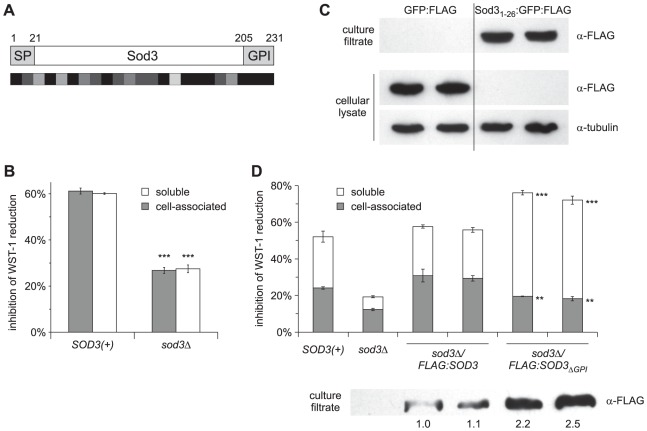
N-terminal and C-terminal signals direct extracellular localization of Sod3. (**A**) Schematic of the Sod3 protein highlighting the predicted signal peptide (SP) and the glycophosphatidyl inositol anchor (GPI) signal motifs. Numbers represent amino acid residues in the Sod3 protein. Shading beneath the Sod3 protein indicates amino acid sequence similarity between G186A, G217B and NAm1 Sod3 proteins ranging from dark (>90% sequence identity) to light (<50% identity). (**B**) Relative Sod3 activity associated with the yeast cell and soluble extracellular fraction. Superoxide dismutase activities were determined by inhibition of superoxide-dependent WST-1 reduction in the presence of 1×10^8^ yeasts (cell-associated) or the corresponding culture filtrate (soluble) of *SOD3(+)* (OSU45) and *sod3Δ* (OSU15) strains (n = 3, each). Inhibition of WST-1 reduction was normalized to reactions in the absence of yeasts or culture filtrate. Asterisks represent significant differences (p<0.001) from *SOD3(+)* samples. (**C**) Determination of the localization of GFP when fused to the N-terminus of Sod3. Extracellular or intracellular GFP localization was determined by α-FLAG immunoblot of culture filtrates or cellular lysates from *Histoplasma* yeast strains expressing FLAG epitope-tagged GFP (GFP:FLAG; OSU88) or GFP with the first 26 amino acids of Sod3 (Sod3_1–26_:GFP:FLAG; OSU102). Cellular lysates were also tested for α-tubulin to demonstrate equal loadings. (**D**) Localization of Sod3 activity after removal of the C-terminal 26 amino acids. Cell-associated and soluble superoxide dismutase activities of *Histoplasma* yeasts were determined using 1×10^8^ intact yeasts or their corresponding culture filtrates, respectively. Samples were collected from *SOD3(+)* (OSU45), *sod3Δ* (OSU15), and yeasts expressing full length Sod3 (*sod3Δ/FLAG:SOD3*; OSU116) or Sod3 lacking the putative GPI signal (sod3Δ/FLAG:*SOD3*_ΔGPI_; OSU117). Results were normalized to uninhibited reactions and plotted as the proportion of total inhibitory activity. Asterisks represent significant difference from full length Sod3 (** p<0.01, *** p<0.001). Relative quantitation of Sod3 in culture filtrates was determined by α-FLAG immunoblot and is indicated numbers below.

Alignment of Sod3 protein sequences derived from the sequenced *Histoplasma* genomes (G186A, G217B, and NAm1) highlights regions of high conservation at the N- and C-termini (Figure 2A). The N-terminus of Sod3 has a predicted signal peptide (amino acids 1–21) that potentially directs the protein into the canonical eukaryotic secretion pathway consistent with Sod3's localization to the extracellular environment [21]. In contrast to the variable sequence identity found throughout the central region of the protein among *Histoplasma* Sod3 orthologs (which includes the core domain shared among enzymes with superoxide dismutase catalytic activity), the C-terminal 24 amino acids are 100% identical suggesting conservation of an important function beyond superoxide dismutase activity. Further inspection of the C-terminus identifies a potential glycophosphatidyl inositol (GPI) attachment signal with a putative omega site at residue 205. GPI signals characterize a number of *Saccharomyces cerevisiae* and *Candida albicans* cell wall proteins in which the GPI anchor is thought to be subsequently cleaved and the protein covalently attached to the cell wall [24], [25]. These two motifs potentially direct secretion of Sod3 from yeast cells and may provide for its association with the yeast cell wall in addition to its observed presence in the soluble culture filtrate.

To determine if a portion of Sod3 is associated with the *Histoplasma* yeast cell wall, yeast cells and their corresponding culture filtrates were tested for superoxide dismutase activity. Addition of cells and culture filtrates to the superoxide generating/WST-1 system showed that both culture filtrates and cells prevent superoxide-dependent reduction of WST-1 (Figure 2B). Compared to the buffer control, cell-associated Sod activity inhibits WST-1 reduction by 61%. A comparable amount of culture filtrate inhibits WST-1 reduction by 60%. Thus, approximately 50% of the total extracellular Sod activity is associated with the cell and 50% is found in the soluble fraction. Sod activity associated with *sod3Δ* cells and their culture filtrates inhibit WST-1 reduction by 26% and 27%, respectively, indicating that the majority of the cell-associated and cell-free Sod activity is attributable to cell-associated and cell-free Sod3 protein.

To determine if the putative N-terminal signal peptide of Sod3 functions as a secretion signal, we tested whether the N-terminus could direct secretion of a normally cytosolic protein. Sequence encoding the first 26 amino acids of Sod3 was fused to a *gfp* coding sequence which had a C-terminal FLAG epitope to allow monitoring of the GFP protein localization by immunoblotting. Expression of *gfp* lacking the N-terminal Sod3 residues causes GFP accumulation in the cytosol of yeast cells as determined by fluorescence microscopy (data not shown) and by immunoblotting of cellular lysates (Figure 2C). On the other hand, GFP protein possessing the Sod3 N-terminal residues is secreted into the culture filtrate and is effectively absent from the cytosolic fraction (Figure 2C). These results demonstrate that the Sod3 N-terminal residues encode a signal peptide that is sufficient to direct protein secretion from yeast cells.

As yeast cells have significant Sod3 activity, we examined the role of the putative GPI signal in mediating cell-association of Sod3. To track the localization of Sod3, sequence encoding the FLAG epitope was inserted into the *SOD3* cDNA at nucleotide 79 of the coding sequence thereby preserving the Sod3 signal peptide for secretion. Downstream of the FLAG epitope, the *SOD3* sequence encoded either Sod3 with the putative GPI attachment signal (nucleotides 79–693 encoding amino acids 27–231) or Sod3 without the GPI signal (nucleotides 79–615 encoding amino acids 27–205). Each construct was transformed into *sod3*Δ yeasts. To determine if deletion of the putative GPI attachment signal reduced cell-association, yeast cells and their corresponding culture filtrates were assayed for their ability to inhibit WST-1 reduction corresponding to superoxide dismutase activity. Expression of FLAG-tagged Sod3 protein yields both cell-free and cell-associated superoxide dismutase activity similar to the ratio observed for *SOD3(+)* yeast cells (Figure 2D). Deletion of the GPI signal from Sod3 significantly decreases cell-associated Sod activity, approaching background levels of *sod3*Δ mutant yeasts (18% and 12% inhibition of WST-1 reduction, respectively). This is consistent with diminished retention of cell-associated Sod3 when the GPI signal is removed.

From this data alone, the possibility that loss of the C-terminal GPI signal causes misfolding of Sod3, either resulting in the protein being degraded within the cell or being transported to the extracellular fraction in an inactive form, cannot be ruled out. To address this, we examined the superoxide dismutase activity in matching culture filtrates from these yeast cells. We found that the decrease in cell-associated Sod3 is accompanied by an increase in soluble Sod3 activity in the culture filtrate (54–57% inhibition of WST-1 reduction compared to 26–27% inhibition by GPI signal-containing Sod3). As further evidence of the redirection of Sod3 from the cell surface to the soluble fraction in the absence of the GPI signal, immunoblotting of culture filtrates shows approximately 2.4-fold more Sod3 protein is released into the culture filtrate without the GPI signal than when the GPI signal is present (Figure 2D). Attempts to directly measure cell-associated Sod3 protein by immunoblotting failed despite treatment of the cell wall fraction with reductants (dithiothreitol), ionic detergent (SDS), or glycanases (zymolyase and chitinase) and combinations of each to release soluble Sod3 from insoluble cell wall material (data not shown). Nonetheless, depletion of cell-associated Sod3 activity and the corresponding increase in Sod3 in the culture filtrate when the GPI signal is removed indicates the GPI signal promotes cell-association of a portion of the total secreted Sod3.

### Sod3 protects specifically against extracellular reactive oxygen

**Figure 3:**
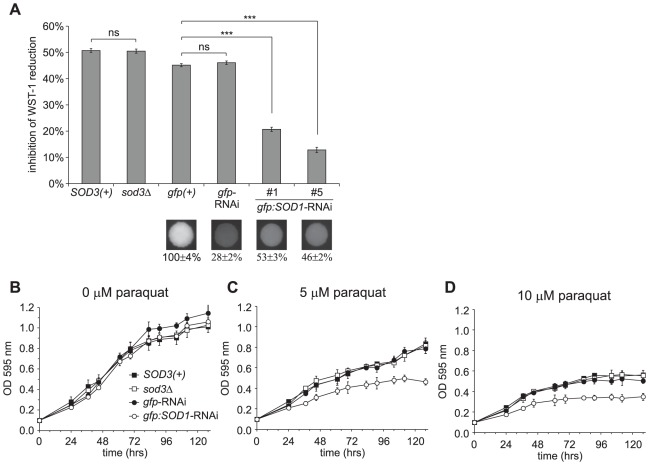
*Histoplasma* Sod3 does not alleviate intracellular oxidative stress. (**A**) Depletion of intracellular superoxide dismutase activity by *SOD1-RNAi* but not by loss of Sod3. RNAi-based depletion of Sod1 was done in a GFP-expressing *Histoplasma* strain (OSU22). GFP fluorescence is shown in colony images of a strain in which *gfp* was not targeted (*gfp(+)*; OSU103), *gfp* alone was targeted (*gfp-RNAi*; OSU104) or two independent isolates in which *gfp* and *SOD1* were co-targeted (*gfp:SOD1-RNAi*; OSU105). Numbers below the images indicate relative GFP fluorescence. Intracellular superoxide dismutase activity was determined by inhibition of WST-1 reduction using 5 µg of cellular lysate protein from *SOD3(+)* (OSU45), *sod3Δ* (OSU15), and the RNAi strains and results plotted relative to uninhibited reactions using 5 µg BSA. Non-significant (ns) and significant (*** p<0.001) differences between *SOD3(+)* and *sod3Δ* or between the *SOD1(+)* strain (*gfp(+)*) and the *gfp*-RNAi or *SOD1*-RNAi strain are indicated above the columns. (**B–D**) Inhibition of yeast growth by increased intracellular reactive oxygen. Liquid growth of *SOD3(+)* (OSU45), *sod3Δ* (OSU15), Sod1-proficient (*gfp-RNAi*; OSU104), and *SOD1-RNAi* (*gfp:SOD1-RNAi*; OSU105) strains was determined by optical density of cultures measured at 595 nm. Intracellular reactive oxygen was increased in yeasts by addition of 5 µM (**C**) or 10 µM (**D**) paraquat. Growth curve points represent the mean optical density of replicate cultures (n = 3).

The extracellular localization of Sod3 predicts Sod3 is produced to combat exogenous but not intracellular superoxide. Indeed, *Histoplasma* yeasts express additional Sod proteins homologous to intracellular superoxide dismutase enzymes and these are more appropriately positioned to detoxify endogenous superoxide such as would be generated from aerobic respiration. The *Histoplasma SOD1* gene encodes a protein homologous to Cu/Zn superoxide dismutase enzymes commonly found in the cytosol of eukaryotes [21]. To determine the specificity of Sod3 and Sod1 for extracellular and intracellular superoxide, respectively, we compared the ability of strains deficient for Sod3 and strains depleted of Sod1 for their ability to survive intracellular superoxide stress. To enable these tests, we depleted Sod1 activity from *Histoplasma* yeasts by RNA interference (RNAi). Depletion of Sod1 function in a GFP-fluorescent RNAi sentinel strain [26] was initially determined by reduced GFP fluorescence caused by co-targeting *gfp* and *SOD1* sequence (Figure 3A). Two independent RNAi lines were created in which the fluorescence of the GFP sentinel is reduced 47%–54%, which is indicative of knock-down of Sod1 function. To confirm depletion of Sod1 function, yeast cellular lysates were tested for superoxide dismutase activity. While deletion of the *SOD3* locus removes extracellular superoxide dismutase activity (Figure 1B), deletion of *SOD3* does not reduce cytosolic superoxide dismutase activity compared to isogenic *SOD3(+)* yeasts (51% and 50% inhibition of WST-1 reduction, respectively; Figure 3A). However, depletion of Sod1 activity by RNAi diminishes cytosolic superoxide dismutase activity compared to the isogenic *gfp*-RNAi and parental *gfp(+)* strains (21% and 13% compared to 46% and 45% inhibition of WST-1 reduction, respectively). Thus, loss of Sod3 specifically affects extracellular but not cytosolic superoxide dismutase activity. Conversely, depletion of Sod1 specifically reduces intracellular superoxide dismutase activity.

Sod1-depleted and Sod3-deficient strains were tested for their sensitivities to intracellular superoxide to ascertain their roles in protecting against intracellular superoxide stress. Yeast cells were grown in liquid culture with increasing amounts of paraquat, which interacts with intracellular redox systems and the mitochondrial respiratory chain to cause increased formation of superoxide [27], [28]. Paraquat treatment of yeast cells causes a dose-dependent decrease in growth as measured by the optical density of yeast cells, indicating paraquat is detrimental to *Histoplasma* cells (Figure 3B–D). *Histoplasma* yeasts are susceptible to paraquat at concentrations of 5 µM and greater, and yeast cells lacking Sod3 show no enhanced sensitivity compared to yeasts producing Sod3 (Figure 3C–D). In contrast, yeast cells depleted of Sod1 show greater sensitivity to paraquat than isogenic cells expressing normal amounts of Sod1. Thus, Sod1 contributes to alleviation of intracellular superoxide stress, but Sod3 does not mitigate intracellular superoxide. These results are consistent with Sod3 specifically functioning in combating exogenous oxidative stress.

**Figure 4:**
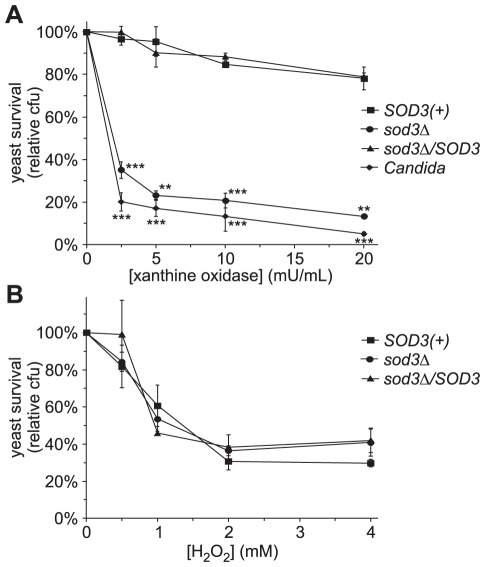
Sod3 protects *Histoplasma* yeast cells from exogenous superoxide in vitro. (**A**) Survival of yeast cells following challenge with superoxide. Yeasts were incubated in increasing amounts of superoxide generated by addition of increasing amounts of xanthine oxidase to hypoxanthine. *SOD3(+)* (OSU45), *sod3Δ* (OSU15), *sod3Δ/SOD3* (OSU49), and *Candida albicans* yeasts were incubated for 4 hours at 37°C after which viable colony forming units (cfu) were determined. Results are plotted as relative yeast survival compared to viable cfu of yeasts incubated in the absence of superoxide (0 mU/mL xanthine oxidase). Results represent the mean ± standard deviations from 3 replicate challenges per strain. Asterisks indicate significant differences (** p<0.01, *** p<0.001) from the *SOD3(+)* strain. (**B**) Sensitivity of *Histoplasma* yeasts to hydrogen peroxide. Increasing amounts of hydrogen peroxide were added to *Histoplasma* yeasts (n = 3 for each strain) at 37°C and the viability of yeasts after 4 hours was determined by enumeration of viable cfu. Results are plotted as relative yeast survival compared to viable cfu of yeasts incubated in the absence of peroxide (0 mM hydrogen peroxide). Data is representative of 3 independent experiments.

To test the role of Sod3 in protecting *Histoplasma* from exogenous superoxide, yeast cells were challenged in vitro with superoxide. To generate superoxide in vitro, graded amounts of xanthine oxidase were added to yeast suspensions in Tris buffer with excess hypoxanthine; the amount of superoxide proportionally increases with the concentration of xanthine oxidase enzyme (Figure S1). Parental *SOD3(+)* yeasts largely survive the in vitro superoxide challenge (Figure 4A). However, yeasts lacking Sod3 are unable to survive superoxide challenge; only 23–35% of *sod3*Δ yeasts survive challenge with superoxide levels at which the Sod3-producing yeasts exhibit greater than 95% survival. Challenge of yeasts with the highest level of superoxide that kills roughly 20% of *SOD3(+)* yeasts, kills over 85% of the *sod3*Δ mutant yeasts. Complementation of the *sod3*Δ mutant restores protection against superoxide challenge to *SOD3(+)* levels. Interestingly, the impaired survival of Sod3-deficient *Histoplasma* yeasts resembles the degree of killing of *Candida albicans* yeasts by superoxide (Figure 4A). These results demonstrate that Sod3 protects *Histoplasma* yeasts from exogenous superoxide.

Superoxide is only one reactive oxygen species with potentially lethal effects on *Histoplasma* yeasts. Spontaneous dismutation of the superoxide that is generated by the xanthine oxidase/hypoxanthine system could also expose yeasts to peroxide stress that could affect yeast cell viability. To determine if peroxide contributes to the enhanced superoxide killing of *sod3*Δ cells, yeasts were challenged in vitro with different concentrations of hydrogen peroxide (0 mM to 4 mM). Increasing the concentration of hydrogen peroxide kills an increasing proportion of *Histoplasma* yeasts (Figure 4B). However, Sod3-expressing and Sod3-deficient yeast have very similar susceptibilities to hydrogen peroxide. These results indicate that peroxide, a potential reactive oxygen derivative of superoxide, does not significantly contribute to the increased killing of *sod3*Δ mutant yeast cells during challenge with superoxide.

### Sod3 protects *Histoplasma* yeast cells from phagocyte-derived reactive oxygen

**Figure 5:**
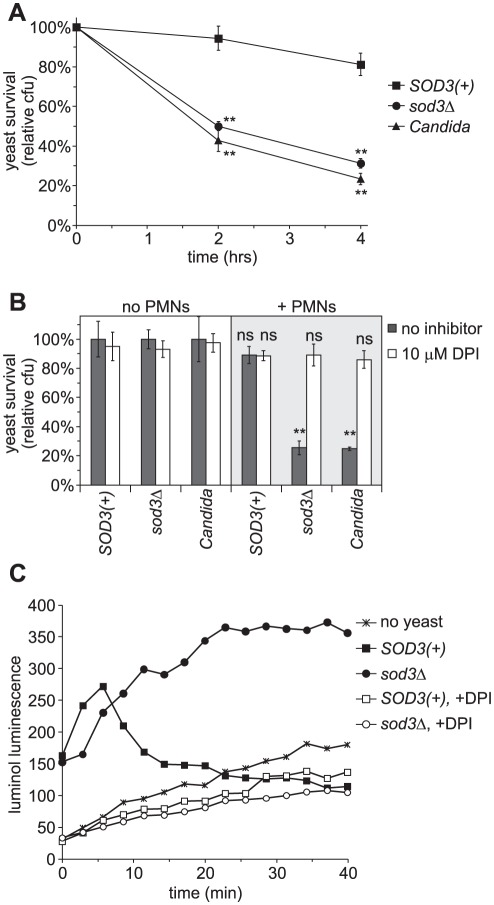
Sod3 protects *Histoplasma* yeasts from PMN-derived reactive oxygen. (**A**) Survival of yeasts after infection of human PMNs. *SOD3(+)* (OSU45), *sod3Δ* (OSU15) and *Candida albicans* yeasts were added to PMNs at a multiplicity of infection (MOI) of 1∶10. Yeast survival was determined by enumeration of viable cfu after 2 and 4 hours of co-incubation of yeasts with PMNs at 37°C. Results are plotted as relative yeast survival (mean ± standard deviation of 3 replicates) compared to viable cfu of yeasts incubated in the absence of PMNs. Significantly decreased survival compared to *SOD3(+)* yeasts is indicated by asterisks (** p<0.01). (**B**) Inhibition of yeast killing by PMNs upon inactivation of the NADPH-oxidase. Yeasts were added to PMNs (+ PMNs) and incubated for 4 hours at 37°C and viable cfu were determined. 10 µM diphenylene iodinium (DPI) was added to some assays to inactivate the NADPH-oxidase. Results indicate relative yeast survival (mean ± standard deviation of 3 replicates) compared to viable cfu of yeast incubated in the absence of PMNs (no PMNs). Significant (** p<0.01) or non-significant (ns) reduction in survival compared to yeast in the absence of PMNs is indicated above the respective columns. (**C**) Reactive oxygen production by PMNs in response to *Histoplasma* yeasts. *Histoplasma* yeasts were added to PMNs at an MOI of 1∶1 in the presence of the luminol ROS-detection reagent and the luminol luminescence measured over time. PMNs and yeasts were co-incubated in the presence (open symbols) or absence (closed symbols) of 10 µM DPI to inhibit the NADPH-oxidase. Data points represent the mean luminescence (n = 3).

The primary source of exogenous reactive oxygen encountered by *Histoplasma* during infection is that produced by host phagocytic cells. Of these cells, PMNs are notorious for their strong oxidative burst in response to pathogens. To test if Sod3 defends *Histoplasma* yeasts from killing by PMNs, *SOD3(+)* and *sod3*Δ mutant yeasts were co-incubated with human PMNs. *SOD3(+)* yeasts largely survive the encounter with PMNs exhibiting 94% and 81% viability after 2 hours and 4 hours, respectively (Figure 5A). Without Sod3, *Histoplasma* yeast cells are efficiently killed by PMNs; only 50% of the *sod3Δ* yeast cells remain viable after 2 hours and viability drops to 31% by 4 hours. As was observed with in vitro sensitivity to superoxide challenge, PMN killing of *sod3*Δ yeast cells mirrors the susceptibility of *Candida* to PMN killing (Figure 5A).

The fungicidal effect on Sod3-deficient yeasts depends on PMN production of reactive oxygen. Suppression of the NADPH-oxidase complex by treatment of PMNs with 10 µM diphenylene iodinium (DPI; [29]) substantially reduces PMN killing of *Histoplasma* and *Candida* yeast cells (Figure 5B). Survival of *sod3*Δ yeasts when co-incubated with PMNs improves from 26% to 89% when the NADPH-oxidase is inhibited, restoring yeast viability to that of Sod3-producing *Histoplasma* yeasts. Impairing NADPH-oxidase function similarly enhances *Candida* survival (Figure 5B). DPI itself has no detrimental effects on yeast cells (Figure 5B) nor does DPI impair PMN viability (Figure S2A, [30]).

The protective effects of DPI or Sod3 on *Histoplasma* viability derive from their ability to decrease toxic reactive oxygen molecules, either by preventing superoxide production or by dismuting PMN-produced superoxide, respectively. To demonstrate this, we measured reactive oxygen levels produced during co-incubation of yeasts and PMNs with luminol (Figure 5C). In the absence of yeast cells, highly reactive PMNs gradually produce reactive oxygen species as indicated by the slow rate of increasing luminol luminescence. Addition of both *SOD3(+)* and *sod3*Δ *Histoplasma* cells stimulates rapid production of reactive oxygen by the PMNs, confirming PMNs react to *Histoplasma* yeasts. While reactive oxygen species persist when *sod3*Δ yeast cells are added, the reactive oxygen quickly disappears in the presence of yeast cells producing Sod3. Addition of DPI abrogates the luminol luminescence triggered by both *SOD3(+)* and *sod3*Δ strains, consistent with its ability to inhibit the NADPH-oxidase. Together these data show that although yeast interaction with PMNs triggers production of fungicidal reactive oxygen, *Histoplasma* Sod3 eradicates the superoxide generated thereby providing for increased yeast survival.

**Figure 6:**
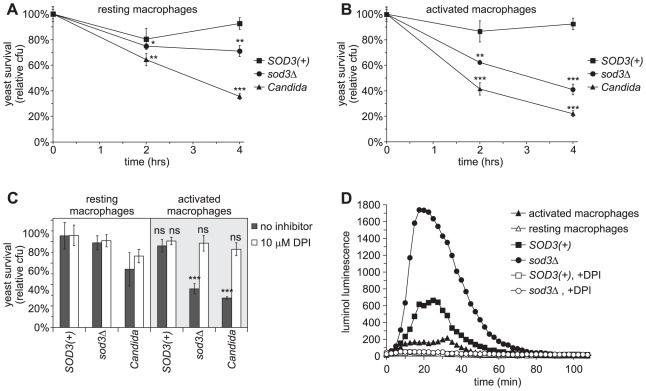
Sod3 protects *Histoplasma* yeasts from ROS produced by activated macrophages. (**A–B**) Survival of yeasts after infection of resting (**A**) or cytokine-activated (**B**) murine macrophages. *SOD3(+)* (OSU45), *sod3Δ* (OSU15) and *Candida albicans* yeasts were added to resident peritoneal macrophages at an MOI of 1∶50. Yeast survival was determined by enumeration of viable cfu after 2 and 4 hours of co-incubation of yeasts with macrophages at 37°C. In (**B**), 10 U TNFα and 100 U IFNγ were added to macrophages 24 hours prior to infection to enhance ROS production. Results are plotted as relative yeast survival (mean ± standard deviation of 3 replicates) compared to viable cfu of yeasts incubated in the absence of macrophages. Significantly decreased survival compared to *SOD3(+)* yeasts is indicated by asterisks (* p<0.05, ** p<0.01, *** p<0.001). (**C**) Prevention of yeast killing by macrophages after inhibition of the NADPH-oxidase. Yeasts were added to resting and to IFNγ/TNFα-activated macrophages and incubated for 4 hours at 37°C in the absence or presence of 10 µM diphenylene iodinium (DPI) and viable cfu were determined. Results indicate relative yeast survival (mean ± standard deviation of 3 replicates) compared to viable cfu of yeasts incubated in the absence of macrophages. Significant (** p<0.01) or non-significant (ns) reduction in survival compared to yeasts in the absence of macrophages is indicated above the respective columns. (**D**) Reactive oxygen production by activated macrophages in response to *Histoplasma* yeasts. *Histoplasma* yeasts were added to resting or activated macrophages at an MOI of 1∶1 in the presence of the luminol ROS-detection reagent and the luminol luminescence measured over time. Macrophages and yeasts were co-incubated in the presence (open symbols) or absence (closed symbols) of 10 µM DPI to inhibit the NADPH-oxidase. Data points represent the mean luminescence (n = 3).

In addition to encountering PMNs, *Histoplasma* also infects macrophages during infection of the host. To explore the role of Sod3 in this interaction, we measured the survival of *Histoplasma* yeast cells following infection of both resting and activated macrophages. 80% and 93% of *SOD3(+)* yeast cells remain viable after infection of resting macrophages at 2 and 4 hours (Figure 6A). The absence of Sod3 results in a small but significant decrease in *sod3*Δ yeast viability compared to *SOD3(+)* yeasts consistent with minimal production of reactive oxygen by resting macrophages in response to *Histoplasma*. When macrophages are activated with IFNγ and TNFα to enhance production of reactive oxygen species, *SOD3(+) Histoplasma* yeasts continue to survive (Figure 6B). However, the viability of *sod3*Δ yeasts in these activated macrophages is considerably reduced with only 62% and 41% surviving at 2 and 4 hours. Macrophages treated with IFNγ, but not TNFα, similarly demonstrate anti-*Histoplasma* activity, but the degree of killing is enhanced by the presence of both cytokines (data not shown). Interaction of *Candida* yeasts with either resting or activated macrophages decreases *Candida* viability (Figure 6A–B).

Since production of superoxide is not the only antimicrobial mechanism in the macrophage arsenal, we tested survival of yeast cells in macrophages that are unable to produce superoxide. Inhibition of the NADPH-oxidase complex with DPI prevents killing of *Histoplasma sod3*Δ and *Candida* yeasts by activated macrophages, indicating that the majority of the macrophage fungicidal activity at 4 hours post-infection requires the production of reactive oxygen compounds (Figure 6C). As with PMNs, treatment of phagocytes with 10 µM DPI does not impair host cell viability (Figure S2B). As independent evidence that the outcome of the interaction between yeasts and macrophages involves oxidative killing, we monitored the macrophage oxidative burst during infection with *Histoplasma* yeasts (Figure 6D). In the absence of fungi, both resting and activated macrophages produce little reactive oxygen. When infected with *Histoplasma* yeasts, resting macrophages produce negligible reactive oxygen, indicating yeasts do not stimulate an oxidative burst in these cells (data not shown). This is not the case with activated macrophages where *Histoplasma* yeasts trigger an initial burst of reactive oxygen within 5 minutes that peaks 10–20 minutes after addition of yeasts (Figure 6D). Considerably less reactive oxygen is detected (65% decrease) if the infecting yeasts produce Sod3 than if the yeasts lack Sod3, consistent with Sod3 destroying superoxide. Thus, activated macrophages produce reactive oxygen species in response to *Histoplasma* yeasts, but yeast-generated Sod3 destroys these reactive compounds and this ROS diminution correlates with enhanced survival of *Histoplasma* in phagocytes.

### Sod3 promotes *Histoplasma* virulence in vivo

**Figure 7:**
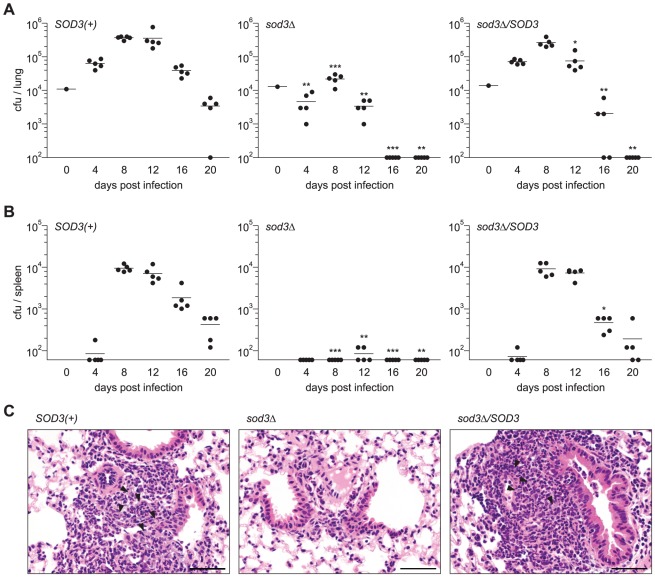
*Histoplasma* virulence in vivo requires Sod3. (**A**) Kinetics of sublethal lung infection by *Histoplasma*. Wild-type C57BL/6 mice were intranasally infected with approximately 1×10^4^ *SOD3(+)* (OSU45), *sod3Δ* (OSU15), or *sodΔ/SOD3* (OSU49) *Histoplasma* yeasts. At 4 day intervals post-infection, the fungal burden in lungs was determined by quantitative platings for *Histoplasma* cfu. (**B**) Kinetics of dissemination following lung infection with *Histoplasma*. At each time point, organs were harvested and the fungal burden in spleen tissue was determined by quantitative platings for cfu. In (**A**) and (**B**), each data point represents cfu counts per organ from an individual animal (n = 5 per time point) and horizontal bars represent the mean fungal burden. Asterisks indicate significant differences at each time point from animals infected with *SOD3(+)* organisms (* p<0.05, ** p<0.01, *** p<0.001). The actual inoculum dose is shown in graphs at day 0. The limit of detection is 100 cfu for lungs and 60 cfu for spleen tissue. (**C**) Inflammation and pathology of lung tissue following *Histoplasma* infection. Wild-type C57BL/6 mice were infected with *SOD3(+)* (OSU45), *sod3Δ* (OSU15), or *sodΔ/SOD3* (OSU49) yeast and at 4 days post-infection, lungs were harvested and sections stained with hematoxylin and eosin. Arrowheads indicate detectable clusters of yeast cells. Scale bars represent 50 µm.

To determine the contribution of Sod3 to *Histoplasma* virulence in vivo, we measured the ability of yeasts lacking Sod3 function to infect murine tissues. As indicators of respiratory and systemic disease, the fungal burden was determined in lung and spleen tissues, respectively, following intranasal delivery of a sublethal inoculum. *SOD3(+)* yeasts infect and replicate within the lung tissue, increasing the fungal burden 400-fold through day 8. The onset of cell-mediated immunity begins to clear *SOD3(+)* yeasts from the lung after day 12 (Figure 7A). With *SOD3(+) Histoplasma* yeasts, dissemination to the spleen occurs by day 4 and rapidly increases in this tissue (Figure 7B). As observed in the lung, the fungal burden begins to clear in the spleen after day 12. In contrast to *SOD3(+)* yeasts, the population of *sod3*Δ yeasts initially decreases within the lung through day 4 without the protective function of Sod3 (Figure 7A). Although there is some expansion of *sod3*Δ yeasts between day 4 and day 8, the number of mutant yeasts barely increase above the inoculum level and then shows rapid clearance starting at day 12. Dissemination of *sod3*Δ yeasts from the lung to the spleen is nearly undetectable, most likely as a consequence of the substantially diminished number of *sod3*Δ yeasts in the lung (Figure 7B). Complementation of the *sod3*Δ mutant by expression of *SOD3* genomic DNA, restores the ability of yeasts to survive and replicate in the lung (Figure 7A), as well as dissemination to the spleen (Figure 7B).

Lung tissue pathology caused by *Histoplasma* infection correlates with lung colonization by Sod3-expressing and Sod3-deficient strains. At 4 days post-infection, lesions are more pronounced in lungs infected with *SOD3(+)* yeasts than with *sod3Δ* yeasts (Figure 7C and Figure S3A). In *SOD3(+)*-infected lungs, more inflammatory foci are present (1–13 per section) with thick collars of inflammatory cells composed of PMNs with fewer alveolar macrophages and lymphocytes (Figure 7C). Yeasts are associated with the inflammatory foci and their numbers correlate with inflammation severity. In contrast, inflammatory foci are rare (0–2 per section) with sparse cellular infiltrates in *sod3Δ*-infected lungs (Figure 7C). Sod3-deficient yeasts are rarely observed consistent with their clearance by the influx of PMNs. Those surviving yeasts that are present are presumably within macrophages. By 8 days post-infection, when *SOD3(+)* fungal burdens are approaching their maximal level, thick collars of inflammatory cells (primarily macrophages and lymphocytes with fewer neutrophils) surround most blood vessels and/or bronchioles (Figure S3B). Interstitial myxedema and congestion is present with inflammation extending into the parenchyma. In *sod3Δ*-infected lungs at 8 days, fewer and less dense inflammatory foci are present. Thus, inflammation severity and tissue pathology, as indicators of disease, closely parallel the fungal burden established by Sod3-expressing and Sod3-deficient yeasts in vivo.

**Figure 8:**
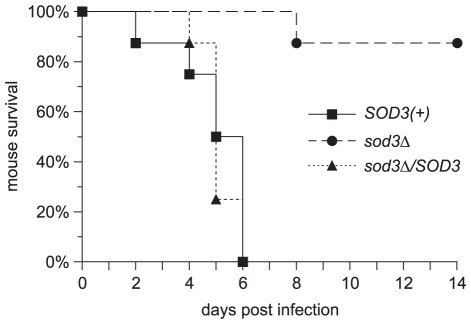
Lethal infection by *Histoplasma* requires Sod3 function. Kinetics of mouse survival after infection with a lethal dose of *Histoplasma* yeasts. Wild-type C57BL/6 mice were intranasally infected with 7×10^6^ *SOD3(+)* (OSU45), *sod3Δ* (OSU15), or *sodΔ/SOD3* (OSU49) *Histoplasma* yeasts (n = 8 per strain). Survival time of mice infected with *sod3Δ* yeasts differs significantly from that of infections with *SOD3(+)* and *sodΔ/SOD3* (p<0.0001).

To determine if establishment of lethal histoplasmosis requires Sod3, mice were inoculated with a lethal dose of *Histoplasma SOD3(+)* and *sod3Δ* yeasts. Mice infected with *SOD3(+)* and Sod3-complemented (*sod3Δ/SOD3*) strains have a median survival time of 5.5 and 5 days, respectively (Figure 8). In contrast, nearly all mice infected with the *sod3Δ* strain survive through two weeks (Figure 8) and appear to have fully recovered from the high inoculum as they show no adverse symptoms and have regained or surpassed their initial body weight (data not shown). Thus, the protective effects of Sod3 are required for *Histoplasma* to establish both lethal and sublethal infections in vivo. The differing fungal burdens, lung pathology, and host survival determined for Sod3-deficient and Sod3-producing strains demonstrate that Sod3 is essential for the full virulence of *Histoplasma* in vivo.

The virulence attenuation due to loss of Sod3 function contrasts with the full virulence of yeasts depleted of the intracellular superoxide dismutase, Sod1. Depletion of Sod1 by RNAi (Figure 3) from *Histoplasma* yeasts does not impair lung infection; the *gfp*-RNAi and *gfp:SOD1*-RNAi strains establish comparable fungal burdens in lungs throughout acute and clearing stages of infection (Figure S4). These results demonstrate that *Histoplasma* virulence specifically requires extracellular (Sod3) superoxide dismutase function.

**Figure 9:**
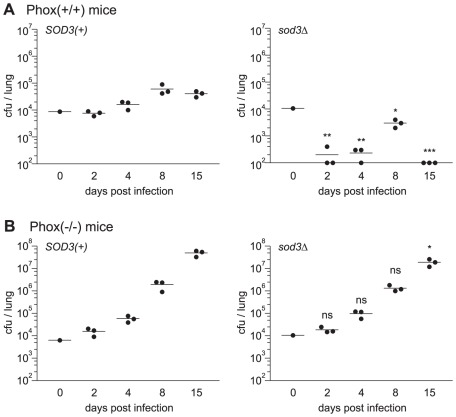
Sod3 facilitates infection through detoxification of host reactive oxygen. Kinetics of sublethal lung infection by *Histoplasma* in animals competent for ROS production (**A**) or animals lacking the NADPH-oxidase function (**B**). Mice were intranasally infected with approximately 1×10^4^ *SOD3(+)* (OSU45) or *sod3Δ* (OSU15) *Histoplasma* yeasts. At 2, 4, 8, and 15 days post-infection, the fungal burden in lungs was determined by quantitative platings for *Histoplasma* cfu. (**A**) Respiratory infection of Phox(+/+) mice isogenic to the p47^phox^ knock-outs. (**B**) Respiratory infection of p47^phox^ knock-out (Phox(−/−)) mice. Each data point represents cfu counts per lung from an individual animal (n = 3 per time point) and horizontal bars represent the mean fungal burden. Non-significant (ns) or significant differences (* p<0.05, ** p<0.01) from animals infected with *SOD3(+)* organisms is indicated above the respective columns. The actual inoculum dose is shown in graphs at day 0. The limit of detection is 100 cfu.

Virulence in the animal model reflects the summation of multiple aspects of the immune response in control of *Histoplasma* yeasts. To clearly define the mechanism of the attenuation of *sod3*Δ yeasts in vivo, we tested the effect of specifically eliminating superoxide production by the host. Infections were established in mice lacking a functional phagocyte NADPH-oxidase complex (Phox) due to mutation of the p47^Phox^ subunit [31]. If the reduced virulence of *sod3*Δ yeasts in vivo results from inability to survive host-produced reactive oxygen, eliminating this host defense mechanism would restore the virulence of *sod3*Δ yeasts. In Phox(+/+) mice, *SOD3(+) Histoplasma* yeasts survive and establish respiratory infection as evidenced by the increasing fungal burdens over the first 8 days (Figure 9A). Although the magnitude of the fungal burdens in the lungs of these Phox(+/+) mice are less than that for experiments in Figure 7 due to different susceptibilities of wild-type mice among vendors, the upward trend in fungal burden is repeated in these mice that are isogenic with the Phox(−/−) mice. Similar to earlier results, at least half of the *sod3*Δ yeasts are killed in Phox(+/+) mice, demonstrating the Sod3 requirement for *Histoplasma* survival (Figure 8A). However, in Phox(−/−) mice unable to generate superoxide, *SOD3(+)* and *sod3*Δ yeasts survive and replicate in lung tissue better than in Phox(+/+) hosts. In the Phox(−/−) hosts unable to produce superoxide, both strains establish similar fungal burdens reaching over 10^6^ cfu in lungs by day 8 (Figure 9B *).* In addition, Phox(−/−) mice are unable to clear the normally sublethal infection and mice become moribund at day 15 with nearly 10^8^ fungal cfu per lung (Figure 9B). Importantly, the kinetics and fungal burdens for *SOD3(+)* and *sod3*Δ yeasts in the Phox(−/−) mice are statistically indistinguishable from each other. These infection data show the overall significance of the role of host-derived reactive oxygen in limiting *Histoplasma* infections. Additionally, they demonstrate that the virulence attenuation of *sod3*Δ yeasts is due to reactive oxygen-dependent killing by the host and further confirms Sod3 promotes *Histoplasma* virulence in vivo by detoxifying host-derived reactive oxygen.

## Discussion

We show in this study that the fungal pathogen *Histoplasma capsulatum* resists damage from antimicrobial ROS and demonstrate that this ability directly contributes to *Histoplasma* virulence. The extracellular superoxide dismutase Sod3 imparts resistance to superoxide since *Histoplasma* strains lacking Sod3 are susceptible to killing by superoxide anion and by macrophages and PMNs that produce ROS. Although the repertoire of phagocyte anti-*Histoplasma* defenses includes non-oxidative mechanisms such as hydrolytic enzymes [32], defensins [32], [33], and reactive nitrogen [6], [34], our use of an NADPH-oxidase inhibitor and Phox(−/−) mutant mice demonstrate that killing of the *sod3Δ* mutant is mediated by superoxide production. The protection of yeasts due to Sod3 mechanistically explains previous studies that show phagocytosis of *Histoplasma* yeasts by PMNs and activated macrophages is accompanied by ROS production, yet the wild-type yeasts remain viable [5], [16]–[19].

Extracellular superoxide dismutases are appropriately positioned to combat host-derived ROS. Unlike peroxide, superoxide is a charged molecule that does not readily cross cellular membranes. This has important consequences for intracellular pathogens. First, the lack of diffusion across membranes maintains higher concentrations of superoxide within the phagosomal lumen. Second, lack of diffusion into the fungal cell cytoplasm requires superoxide defense mechanisms to be located extracellularly. The *Histoplasma* Sod3 protein has an N-terminal signal sufficient to direct it into the secretory pathway and a C-terminal signal that promotes association with the cell surface. Cell-associated Sod3 may be covalently linked to the cell wall as has been shown for some yeast cell wall proteins with GPI signals [24], [25]. Consistent with this, we have been unable to recover soluble Sod3 from cell wall preparations after treatment with reducing agents, anionic detergents, and glycanases (unpublished data). It is unknown if the portion of Sod3 protein that is not associated with yeast cells represents protein previously located at the cell surface and subsequently shed or whether it is protein secreted directly into the extracellular milieu. We suspect soluble Sod3 reflects insufficient retention on the cell since deletion of the C-terminal GPI signal shifts Sod3 from association with the cell into the soluble fraction. Deletion of the GPI signal did not completely prevent association of Sod3 with the cell, suggesting the existence of other unidentified cell-association signals. Regardless, both soluble and cell-associated Sod3 are appropriately located extracellularly in order to detoxify superoxide in the phagosomal lumen.

Our study is the first to demonstrate *Histoplasma* produces extracellular superoxide dismutase activity and identifies its source as Sod3. These results differ from earlier studies that failed to show *Histoplasma* yeasts could dismute exogenously generated superoxide in vitro [10], [11]. A number of experimental differences likely account for this discrepancy. In the earlier in vitro studies, nearly 4 times more superoxide were generated and 10-fold less yeast cells were used as the potential superoxide dismutase source. In the current study, the use of a more sensitive detection reagent for superoxide (WST-1; [23], [35]) allowed us to generate less superoxide which did not overwhelm the dismutase activity of the number of yeast cells used in our assays. The deletion of *SOD3* and the corresponding loss of extracellular superoxide dismutase activity provide further evidence of the ability of Sod3-producing *Histoplasma* yeasts to destroy exogenous superoxide. While most studies have agreed that *Histoplasma* yeasts trigger ROS release from PMNs [5], [13], [16]–[19], studies failing to detect ROS production [11] may have misinterpreted the results as lack of ROS stimulation instead of rapid destruction of ROS. Our results show that in the presence of efficient superoxide scavenging by Sod3, phagocyte ROS levels rapidly return to baseline by 10 to 15 minutes resulting in a narrow window for detection (Figure 5C).

The susceptibility of *sod3Δ* yeasts to superoxide challenge in vitro and their attenuated virulence show superoxide is one of the major toxic oxidative compounds against which *Histoplasma* must defend in vivo. Although superoxide can be dismuted to peroxide, either through spontaneous or Sod3-catalyzed dismutation, peroxide is not the toxic form of reactive oxygen responsible for damaging the *sod3Δ* yeasts. As evidence of this, we show that yeasts lacking Sod3 function are killed by superoxide, but do not exhibit any increased sensitivity to peroxide (Figure 4A–B). Lack of peroxide sensitivity may result from efficient destruction of peroxide by *Histoplasma* yeasts since they also express an extracellular catalase [36]–[38]. As an uncharged molecule, peroxide can also diffuse into the yeast cell cytoplasm where intracellular scavenging enzymes may also protect yeasts from oxidative damage from peroxide. Alternatively, yeast viability may rely on essential target molecules that are attacked specifically by superoxide but not peroxide. The impact of exogenous peroxide and the role of *Histoplasma* peroxide defenses await further genetic tests with yeasts lacking catalase antioxidant factors.

The ability to tolerate or destroy host-generated reactive oxygen is a fundamental characteristic of successful microbial pathogens. Extracellular dismutation of superoxide is a capability shared by *Histoplasma* and other intracellular pathogens of macrophages including *Mycobacterium tuberculosis* [39], *Salmonella* spp. [40], [41], and *Francisella tularensis* [42].. A past study demonstrated that the resistance of fungal pathogens to killing by PMNs distinguishes primary from opportunistic pathogens; despite triggering ROS release from PMNs, *Histoplasma*, *Blastomyces*, and *Paracoccidioides* survived whereas *Candida* and *Aspergillus* cells were efficiently killed [19]. Genome analyses of the dimorphic fungal pathogens *Blastomyces*, *Paracoccidioides*, and *Coccidioides* identify proteins homologous to *Histoplasma* Sod3 and these proteins also have predicted N-terminal signal peptides (data not shown). This suggests that, like Sod3, they are extracellular superoxide dismutases that could protect these fungal cells from ROS release during interaction with host cells. The production of extracellular superoxide dismutases thus appears to be a defining characteristic of primary pathogens that are not effectively controlled by the innate immune system. Extracellular superoxide dismutation typifies intracellular pathogens in particular as the pathogenesis of these microorganisms involves engagement of and internalization into superoxide-producing phagocytes.

A number of plant and human pathogenic fungal species express multiple superoxide dismutases, which have been postulated to impart virulence potential. However, the majority of the identified Sods are intracellular enzymes (cytosolic or mitochondrial) and their loss causes sensitivity to intracellular oxidative molecules. This suggests that the in vivo virulence attenuation of fungi with deleted intracellular SOD genes may stem from their impaired ability to alleviate superoxide stress arising from altered metabolism imposed by growth in the host rather than insufficient detoxification of host-derived superoxide. For example, *Cryptococcus gattii* Sod1 and Sod2, *Cryptococcus neoformans* Sod1 and Sod2, and *Candida albicans* Sod1, Sod2, and Sod3 are all intracellular Sods. Deletion of their encoding genes reduces fungal virulence in vivo but also increases sensitivity to pharmacologically-induced intracellular oxidative stress or growth on respiration-requiring media [43]–[46]. *Cryptococcus neoformans sod1Δ* mutants survive and replicate within macrophages albeit at a slightly slower rate also consistent with Sod1 functioning in reducing metabolic rather than host-produced oxidative stress [47]. Whether virulence attenuation in these mutants arises from failure to defend against host-produced oxidative stress awaits testing in hosts unable to produce superoxide. Data for the involvement of superoxide dismutases in fungal infection of plants is based primarily on correlations with *SOD* gene transcription during fungus-plant interaction [48]–[51], but genetic evidence of the role of superoxide dismutase is largely lacking or contradictory in the case of *Botrytis* Sod1 [52], [53]. The results of our study using *Histoplasma* strains deficient in Sod1 and Sod3 demonstrate the spatial specificity of the Sod1 and Sod3 superoxide dismutases for internal and external (i.e., host-derived) superoxide, respectively. Only growth of the Sod1-depleted strain, but not Sod3-deficient yeasts, is impaired when intracellular superoxide levels are increased (Figure 3). Conversely, only Sod3-deficient but not Sod1-depleted yeasts are attenuated in virulence in vivo (Figure 7 and Figure S4). Furthermore, the restoration of *Histoplasma sod3Δ* mutant virulence in mice unable to produce superoxide conclusively shows that Sod3 functions in detoxification of superoxide generated by the host.

However, pathogenic potential is not solely attributable to extracellular Sods and their ability to destroy toxic ROS. For example, the opportunistic fungal pathogen *Candida albicans* expresses at least two extracellular superoxide dismutases (Sod4 and Sod5) that are induced in response to oxidative stress and are required for full virulence in mice [54], [55]. Conflicting results exists as to whether Sod5 affords some protection to *Candida* against superoxide-dependent killing by phagocytes in vitro [54]–[56], and one study shows Sod5 affects sensitivity to miconazole-induced intracellular ROS [57]. Nonetheless, *Candida* is still effectively controlled by innate immune cells. In short term viability assays, we found PMNs kill nearly 80% of *Candida* yeasts (Figure 5A) suggesting *Candida* Sod4 and Sod5 proteins are insufficient to protect against the levels of superoxide generated by PMNs. Resting and activated macrophages similarly killed *Candida* to a large extent in our study (Figure 6A–B). Interestingly, *Histoplasma* yeasts lacking Sod3 closely resemble *Candida* yeasts in their susceptibility to in vitro superoxide and to phagocyte-derived ROS, showing *Histoplasma* Sod3 contributes to the greater resistance of *Histoplasma* to phagocyte killing and consequently to *Histoplasma's* ability to cause disease in immunocompetent hosts.

In addition to detoxification of ROS, the ability to minimize stimulation of the phagocyte respiratory burst also contributes to the pathogenic potential of fungi. Similar to *Candida*, *Histoplasma* stimulates ROS production by PMNs and activated macrophages (Figure 5C, Figure 6D, and [5], [12], [13], [56]). However, the level of ROS release triggered by *sod3Δ* mutant *Histoplasma* yeasts is less than that caused by other phagocyte stimuli such as phorbol myristate acetate and zymosan (data not shown). This may result from decreased phagocyte recognition of *Histoplasma* yeasts since the α-glucan polysaccharide in *Histoplasma* cell walls masks cell wall β-glucans from the phagocyte β-glucan receptor Dectin-1 [58]. On the other hand, *Candida* cell wall molecules are recognized by multiple pattern recognition receptors [59] and Dectin-1 recognition of *Candida* enhances ROS production by phagocytes [60]–[63].

Despite the ability of *Histoplasma* Sod3 to destroy fungicidal superoxide, host-generated ROS provides some limited control of *Histoplasma* during the innate immune response and is critically important for clearance of *Histoplasma* upon activation of immune cells. IFNγ treatment of macrophages primes host cells for ROS production in response to *Histoplasma* yeasts [64] and addition of TNFα further enhances ROS generation [12], [13]. Pre-treatment of macrophages with levels of IFNγ and TNFα used in our study enhance ROS production in response to stimuli while keeping nitric oxide levels relatively low [65]. In vitro, activation of macrophages can variably inhibit *Histoplasma* yeasts [13], [64], [66]–[68] while our results indicate Sod3 protects *Histoplasma* yeasts against activated macrophage ROS, at least in short term assays (up to 4 hours).

Rapid proliferation of both Sod3-producting and Sod3-deficient *Histoplasma* in the lungs of mice lacking NADPH-oxidase function highlights the critical importance of host ROS. Phox(+/+) mice, compared to Phox(−/−) mice, restrict even SOD3(+) *Histoplasma* yeasts, indicating the involvement of oxidative defense mechanisms . While this restrains the infection to some degree, the innate response is unable to clear the infection. Our results indicate that Sod3 function is a major factor in protecting the yeasts from innate immune cells and in promoting histoplasmosis (Figure 7C, Figure 8, and Figure S3). Consistent with this early involvement of ROS, yeasts lacking Sod3 function are rapidly killed during the early phases of infection (Figure 7A and Figure 9A). Nonetheless, the *sod3Δ* yeasts are not entirely cleared and in fact show some proliferation of the fungal burden in lungs between days 4 and 8 (Figure 7A and Figure 9A). We suspect resting macrophages, the infection of which by *Histoplasma* does not result in significant ROS production (Figure 6, and [5], [10]–[12]), provide a temporary refuge for Sod3-deficient yeast. However, influx of CD4^+^ T-cells and the corresponding increase in IFNγ in lungs after day 7 [69] promotes clearance of *sod3Δ* yeasts through activation of macrophages and enhancement of oxidative killing. Cytokine activation of macrophages in culture and their ability to kill *sod3Δ* yeasts support this model (Figure 6B). Thus, Sod3 is an essential virulence factor that protects *Histoplasma* yeasts from killing by ROS produced by the host, particularly during the innate immune response to infection.

## Materials and Methods

### Ethics statement

This study was carried out in accordance with The National Research Council's Guide for the Care and Use of Laboratory Animals, Public Health Service Policy on Humane Care and Use of Laboratory Animals, and AAALAC accreditation guidelines. The protocol was approved by the Ohio State University Institutional Animal Care and Use Committee (protocol number: 2007A0241). Animal procedures were performed under anesthesia as described below and all efforts were made to minimize suffering. Human cells were obtained from healthy volunteers after obtaining HIPAA research authorization and written informed consent in accordance with the Declaration of Helsinki. The human subjects protocol was reviewed and approved by the Ohio State University Biomedical Sciences Institutional Review Board (protocol number 2008H0242) under Ohio State University's Office for Human Research Protections (Federalwide Assurance number: 00006378).

### Fungal strains and culture

*Histoplasma capsulatum* strains used in this study were derived from the wild-type strain G186A (ATCC 26027) and are listed in Table 1. *Histoplasma* yeasts were grown in *Histoplasma*-macrophage medium (HMM; [70]) or modified 3M medium (85 mM NaCl, 1 mM K_2_HPO_4_, 20 mM HEPES, 1.5% glucose, 15 mM (NH_4_)_2_SO_4_, 1 mM Mg_2_SO_4_, 0.2 mM CaCl_2_, 15 mM glutamate, 350 µM cysteine at pH 7.0; [70]) at 37°C. Liquid cultures were continuously shaken (200 rpm) until late log/early stationary phase unless otherwise indicated. For growth of uracil auxotrophs, media was supplemented with uracil (100 µg/mL). Growth rate and growth stage of strains were determined by measurement of liquid culture turbidity at 595 nm. In some experiments media was supplemented with paraquat (856177, Sigma) to a final concentration of 5 µM and 10 µM to artificially raise the intracellular levels of superoxide. For growth on solid media, HMM was solidified with 0.6% agarose and supplemented with 25 µM FeSO_4_. *Histoplasma* yeasts were transformed with linear plasmids by electroporation [71] and plated on solid HMM to select Ura^+^ transformants. For experiments with defined numbers of yeasts, clumps of cells were removed by centrifugation (1 minute at 50 rcf). For precise enumeration, yeast cells were counted using a hemacytometer. *The cap1Δ/cap1Δ Candida albicans* strain (used to prevent filamentous growth of *Candida albicans* which would confuse accurate enumeration of cfu; [72]) was grown in liquid YPD medium or on YPD solidified with 2% agar. *Candida* cells were enumerated by hemacytometer.

**Table 1:** *Histoplasma capsulatum* strains.

Strain^a^	Genotype	Other Designation
WU8^b^	*ura5-32Δ*	
OSU13	*ura5-32Δ sod3-3Δ::hph*	*sod3Δ*
OSU15	*ura5-32Δ sod3-3Δ::hph/pCR468 [URA5, gfp:FLAG]*	*sod3Δ*
OSU22^c^	*ura5-32Δ zzz::pCR482 [hph, gfp]*	
OSU45	*ura5-32Δ/pCR468 [URA5, gfp:FLAG]*	*SOD3(+)*
OSU49	*ura5-32Δ sod3-3Δ::hph/pBY09 [URA5, SOD3]*	*sod3Δ/SOD3*
OSU88	*ura5-32Δ/pCR468 [URA5, gfp:FLAG]*	GFP:FLAG
OSU102	*ura5-32Δ/pCR508 [URA5, SOD3_1–78_:gfp:FLAG]*	Sod3_1–26_:GFP:FLAG
OSU103	*ura5-32Δ zzz::pCR482 [hph, gfp]/pCR464 [URA5]*	*gfp(+)*
OSU104	*ura5-32Δ zzz::pCR482 [hph, gfp]/pCR473 [URA5, gfp-RNAi]*	*gfp-RNAi*
OSU105	*ura5-32Δ zzz::pCR482 [hph, gfp]/pCR579 [URA5, gfp:SOD1-RNAi]*	*gfp:SOD1-RNAi*
OSU116	*ura5-32Δ sod3-3Δ::hph/pCR601 [URA5, SOD3_1–78_:FLAG:SOD3_79–693_]*	*sod3Δ/FLAG:SOD3*
OSU117	*ura5-32Δ sod3-3Δ::hph/pCR602 [URA5, SOD3_1–78_:FLAG:SOD3_79–615_]*	*sod3Δ/FLAG:SOD3_ΔGPI_*

a all strains were constructed in the G186A (ATCC# 26027) background.

b uracil auxotroph of G186A (Marion CM, et al., 2006 [78]).

c GFP sentinel RNAi background (Edwards, et al. 2011 [26]).

### Generation of the *sod3Δ* mutant, *SOD3*-complemented, and *SOD1*-RNAi strains

WU8 *Histoplasma* yeasts were transformed with the *URA5(+)* plasmid pBY06 which contains a hygromycin resistance gene flanked by 2 kb of sequence upstream and 2 kb of sequence downstream of the *SOD3* coding sequence. Hygromycin-resistant transformants were grown in liquid HMM with uracil and 150 µg/mL hygromycin B, diluting the culture 20-fold when stationary phase was reached. 3 passages in liquid culture were performed after which yeast cells were plated on solid HMM containing uracil, 150 µg/mL hygromycin B, and 1 mg/mL 5-fluororotic acid (5-FOA) to counter-select retention of the knock-out allele episomal molecule. Hygromycin-resistant, 5-FOA-resistant colonies were picked and screened for the loss of the *SOD3* CDS by PCR. For complementation, the *SOD3* gene was amplified from wild-type G186A genomic DNA by PCR and cloned into pCR468 replacing the *gfp* transgene. The pCR468 vector contains the histone-2B (*H2B*) promoter for high level constitutive expression of transgenes. The *sod3Δ* mutant was transformed either with the *gfp*- (pCR468) or *SOD3*-expression vector (pBY09) and Ura^+^ transformants selected to create uracil-prototrophic strains. An isogenic strain with the wild-type *SOD3* locus was also created by transformation of WU8 with the *gfp*-expression vector (pCR468).

Depletion of Sod1 was achieved using RNAi. Nucleotides −12 to 787 of the *SOD1* CDS were amplified by PCR and two copies cloned in inverse orientation into the RNAi *gfp*-sentinel vector pCR473 [26]. Linearized plasmids were transformed into OSU22, a WU8-based strain that expresses a *gfp* transgene. Ura^+^ transformants were screened for silencing of the GFP sentinel using a modified UV transilluminator [73]. Transformants with significant loss of GFP fluorescence were selected for analysis of intracellular superoxide dismutase activity as described below.

### Preparation of *Histoplasma* culture filtrate and cellular lysate samples

*Histoplasma* yeasts were grown to late log/early stationary phase in modified 3M medium. Yeast cells were removed by centrifugation (5 minutes at 5000 rcf) and supernatants were filtered through 0.2-µm pore polyethersulfone (PES) membranes. Culture filtrates were concentrated and proteins exchanged into PBS buffer by stir-cell based ultrafiltration using 10 kDa molecular weight cut off PES membranes (Millipore) or centrifugal devices (Sartorius Stedim Biotech). Culture filtrate protein concentrations were determined by DC Lowry assay using ovalbumin as the protein standard for comparison. Concentrated culture filtrates were treated with 50 mM diethyldithiocarbamate (DDC) for 30 minutes to remove Cu^++^ ions. DDC was removed by dialysis against Tris-buffered saline (TBS) before assaying for superoxide dismutase activity. Cu^++^ ions were replenished by incubation of Cu^++^-depleted samples with 50 mM CuSO_4_ for 30 minutes and subsequent dialysis against TBS.

Cellular lysates were prepared by collecting yeast cells from liquid 3M cultures by centrifugation (5 minutes at 5000 rcf). Cytosolic material was liberated from yeast cells by beating the yeasts with 400 µm-diameter glass beads in PBS containing a protease inhibitor cocktail (P2714, Sigma). Cellular lysate protein concentrations were determined by DC Lowry assay using bovine serum albumin as the protein standard.

### Superoxide dismutase assay

Detection of superoxide dismutase activity was based on depletion of superoxide as determined by the superoxide-dependent reduction of the water soluble tetrazolium dye WST-1 (Dojindo Molecular Technologies, [23], [35]). Superoxide was generated in vitro from hypoxanthine using xanthine oxidase (X4500, Sigma). Reactions were performed in a solution consisting of 50 mM Tris pH 8.0, 100 µM hypoxanthine, 5 mU/mL xanthine oxidase, and 100 µM WST-1 to which variable amounts of *Histoplasma* samples were added. WST-1 reduction over time at 25°C was monitored by absorbance at 438 nm using a Synergy 2 plate reader (BioTek). The inhibition of WST-1 reduction was determined by subtracting the value of WST-1 reduction in each sample from that of reactions with buffer alone (corresponding to 100% WST-1 reduction). For assays not comparing cell-associated and soluble activities, culture filtrate samples were normalized to 5 µg total protein in a total volume of 200 µL and the kinetics of WST-1 reduction determined. For experiments comparing cell-associated and soluble superoxide dismutase activity, 1×10^8^ cells (estimated by OD_600_ readings without removal of clumped yeast) were collected by centrifugation and resuspended in a volume of PBS equal to the volume of culture filtrate removed. Soluble superoxide dismutase samples were obtained using the volume of culture filtrate equivalent to that of 1×10^8^ *Histoplasma* yeast cells. Cell-free samples were concentrated and buffer-exchanged into PBS by ultrafiltration. Equivalent proportions of cells or culture filtrates were added to the superoxide generating system in a final volume of 400 µL. After 12 minutes with shaking (1000 rpm), samples were centrifuged to remove cells, 200 µL clarified sample recovered and the WST-1 reduction determined by end-point absorbance at 438 nm. The proportion of superoxide dismutase activity in culture filtrate or cell-associated samples was determined as the fraction of total inhibition of WST-1 reduction relative to the respective buffer controls.

### Construction and analysis of strains expressing variant Sod3 proteins

Nucleotides 1 to 78 of the *SOD3* CDS, encoding the first 26 amino acids of the Sod3 protein, were amplified by PCR and inserted in frame in front of *gfp* with a C-terminal FLAG epitope. Constructs were cloned behind the *Histoplasma H2B* promoter in a *URA5(+)* expression vector (pCR468) to make vector pCR508. Linearized pCR508 and pCR468 plasmids were transformed into *Histoplasma* strain WU8. Culture filtrates and yeast cells were collected from liquid cultures of Ura^+^ transformants. 5 µg of total culture filtrate and cellular lysate proteins were screened for the FLAG epitope by immunoblotting with an anti-FLAG antibody (F1804, Sigma) and for α-tubulin with the anti-tubulin antibody (B512, Sigma).

For construction of Sod3 proteins truncated from the C-terminus, a nucleotide sequence encoding the FLAG epitope was inserted before nucleotide 79 of the *SOD3* CDS. Downstream *SOD3* sequence ended at either nucleotide 615 or 693 of the cDNA. Fragments were amplified and spliced together by PCR. Fusion proteins were directed into the secretory pathway by insertion of sequence encoding the Sod3 signal peptide (nucleotides 1 to 78, encoding the first 26 amino acids) at the 5′ end. Constructs were cloned into the *Histoplasma H2B*-promoter transgene expression vector (pCR468) to generate plasmids pCR601 and pCR602. Linearized plasmids were transformed into OSU13 and Ura^+^ transformants assayed for cell-associated and cell-free soluble superoxide dismutase activities. Statistical significance was determined by one-tailed Student's t-tests. Immunoblotting for the FLAG epitope in culture filtrates was performed by deglycosylating culture filtrate proteins with PNGaseF (NEB) for at least 12 hours prior to separation of proteins by SDS-PAGE and immunoblotting with the anti-FLAG antibody. Immunoblot signals were quantified using ImageJ software (http://rsbweb.nih.gov/ij/).

### Determination of susceptibility to superoxide

For determination of superoxide susceptibility, *Histoplasma* or *Candida* yeast cells were collected from log-phase liquid cultures and 1×10^5^ cells were incubated in a superoxide-generating system consisting of 50 mM Tris pH 8, 100 µM hypoxanthine, and increasing amounts of xanthine oxidase (X4500; Sigma) in a total volume of 500 µL in wells of a 24-well tissue culture plate. Yeasts were incubated for 4 hours at 37°C with shaking (200 rpm) in a humidified chamber. After incubation, yeasts were removed and serial dilutions plated on solid media to determine viable fungal colony forming units (cfu). For determination of peroxide susceptibility, 1×10^5^ yeast cells were incubated in HMM with increasing amounts of hydrogen peroxide (0 to 4 mM). Aliquots were removed at 4 hours and serial dilutions plated on solid media to determine viable cfu. The concentration of the hydrogen peroxide stock solution was determined immediately before use by spectrophotometric absorbance at 240 nm [74]. Survival was statistically compared between strains using one-tailed Student's t-tests.

### PMN isolation and infection

PMNs were isolated from human blood samples obtained by venipuncture of healthy volunteers [75], [76]. 10 mL venous blood were collected into syringes containing 250 U heparin and diluted back 2-fold in 0.9% saline solution. 10 mL of blood suspension was layered onto 4.5 mL Ficoll-Paque PLUS and PMNs were recovered by density sedimentation (40 minutes at 400 rcf at 18°C). Red pellets were harvested and suspended in 6 mL of 0.9% saline before the addition of 6 mL of 3% Dextran_500_. Tubes were gently mixed and incubated at 4°C for 20 minutes. The upper layer was transferred to a new tube, and cells were collected by centrifugation (15 minutes at 800 rcf at 4°C). Residual erythrocytes were lysed by suspending pellets in cold H_2_O and gently mixing for 25 seconds before restoring isotonicity by adding an equal volume of 1.8% saline solution. Recovered cells were washed and suspended in Hanks buffered saline solution (without Mg^++^ or Ca^++^) and cells enumerated by hemacytometer. Cell viability was determined using trypan blue. Autologous serum was prepared from separate blood samples following coagulation. PMNs were seeded into 96-well tissue culture plates at 2×10^5^ cells per well in DMEM with 10% autologous serum and cells were allowed to adhere for 20 minutes at 37°C in 5% CO_2_/95% air. For infection, 2×10^4^ *Histoplasma* or *Candida* yeast cells were added to the PMNs. PMNs and fungal cells were co-cultured at 37°C in 5% CO2/95% air. For inhibition of the NADPH-oxidase, 10 µM diphenylene iodinium (DPI;43088, Sigma) was added to PMNs 20 minutes before infection and maintained throughout the co-culture. At specified times following infection, the culture medium was removed and PMNs lysed by the addition of sterile water and thoroughly scraping wells with a pipette tip. Cell lysates were combined with the removed culture medium and serial dilutions plated to determine viable fungal cfu counts. Yeast survival was compared using one-tailed Student's t-tests. Parallel cultures of PMNs, with and without DPI, were used to score PMN viability during the course of the assay. PMN viability was determined by exclusion of trypan blue. 100 cells were scored for each of seven fields by brightfield microscopy (for a total of 700 cells). For luminescence assays PMNs were collected and seeded at 2×10^5^ cells per well in 96-well plates. To measure production of ROS, the medium was replaced with luminescence buffer (115 mM NaCl, 5.9 mM KCl, 1.2 mM MgCl_2_, 1.2 mM NaH_2_PO_4_, 2.5 mM CaCl_2_, 50 µM luminol, 25 mM NaHCO_3_, 10 mM glucose at pH 7.4) containing 2×10^5^ yeasts. In some assays 10 µM DPI (diphenylene iodinium) was also added to inhibit the NADPH-oxidase. Luminescence of wells was read every 2 minutes at 37°C using a Wallac Victor plate reader.

### Macrophage isolation and infection

Unelicited peritoneal macrophages were obtained by peritoneal lavage of C57BL/6 mice (NCI) according to standard procedures [77]. Following euthanasia, 10 mL PBS were injected into the peritoneum and the peritoneum gently massaged for 1 minute. Peritoneal cells were recovered by aspiration using a 10 mL syringe with an 18-gauge needle inserted into a small incision. Peritoneal cells were collected from the lavage fluid by centrifugation (10 minutes at 700 rcf at 4°C). Cells were resuspended in DMEM with 10% fetal bovine serum and enumerated by hemacytometer. Macrophages were recovered by adherence to plastic for 12 hours and removal of non-adherent cells. 1×10^5^ macrophages were seeded into each well of a 96-well tissue culture plate. 100 U murine IFNγ and/or 10 U murine TNFα (Biolegend) were added to wells for 24 hours at 37°C in 5% CO_2_/95% air for activation of macrophages. For infection, 2×10^3^ *Histoplasma* or *Candida* yeast cells were added to the macrophages. Macrophages and yeast cells were co-cultured at 37°C in 5% CO_2_/95% air. In some wells, 10 µM DPI was added to inhibit the NADPH-oxidase. At incremental times post-infection, the culture medium was removed and macrophages lysed by addition of water. The lysate and removed culture medium were combined and serial dilutions plated on solid media for enumeration of viable fungal cfu. Yeast survival was compared by one-tailed Student's t-tests. Parallel cultures of macrophages, with and without DPI, were used to score macrophage viability during the course of the assay. Macrophage viability was determined by exclusion of trypan blue. 100 cells were scored for each of seven fields by brightfield microscopy (for a total of 700 cells). For luminescence experiments macrophages were harvested as described above and 1×10^5^ macrophages were added to each well of a 96-well plate. ROS production was determined by replacing the medium with luminescence buffer containing 1×10^5^ yeasts. Luminescence of wells was measured every 2 minutes at 37°C.

### In vivo virulence determination

C57BL/6 mice (NCI or Harlan) were infected with *Histoplasma* yeasts by intranasal delivery of 1×10^4^ to 2×10^4^ yeast cells. *Histoplasma* yeast cells were collected from exponentially growing liquid cultures and enumerated by hemacytometer. Actual cfu in the inocula were determined by plating serial dilutions. At 4, 8, 12, 16, and 20 days post infection, mice were euthanized and lungs and spleens collected. Lung and spleen tissues were homogenized in 5 mL and 3 mL HMM, respectively, and serial dilutions of the homogenates plated on solid HMM to determine the fungal burden in each organ. For determination of virulence in mice lacking the phagocyte oxidase, p47 knock-out mice (line 4227, Taconic) or isogenic wild-type mice (line C56BL/6N, Taconic) were infected intranasally with 1×10^4^ to 2×10^4^ *Histoplasma* yeast cells and tissues harvested at 2, 4, and 8 days post-infection. To prevent bacterial infections during the assay time course, 0.2 mg/mL enrofloxacin (Bactrim) and 0.3 mg/mL tetracycline were added to the drinking water and mice were housed on non-abrasive Alpha-dri bedding (Shepherd Specialty Papers). Mean cfu counts were compared between infections by one-tailed Student's t-test for statistical significance.

For histology analyses, mice were intranasally infected with 1×10^4^ to 2×10^4^ yeast cells. After 4 and 8 days, lungs were removed, perforated with a 25-gauge needle, and fixed with 5% formalin in phosphate buffered saline. Lung sections were stained with hematoxylin and eosin and examined and interpreted by a board-certified pathologist (Comparative Pathology and Mouse Phenotyping Facility, Ohio State University College of Veterinary Medicine).

For mouse survival assays, mice were intranasally infected with 7×10^6^ *Histoplasma* yeasts. Mice were monitored daily for survival, weight loss, and symptoms of disease. Mice were determined to be moribund if they demonstrated extreme lethargy, lateral recumbence, or greater than 25% reduction in weight. Statistical analyses were performed using the log-rank test.

## Supporting Information

Figure S1 **Increasing amounts of xanthine oxidase increase the amount of superoxide produced.** (**A**) Production of superoxide by increasing amounts of xanthine oxidase (XOD) with hypoxanthine. A two-fold dilution series of xanthine oxidase was added to 100 mM hypoxanthine and the superoxide produced was detected by reduction of WST-1. WST-1 reduction was monitored over time by absorbance at 438 nm. Data points represent the mean value of triplicate samples. (**B**) Linear relationship between the amount of xanthine oxidase and the superoxide produced. The amount of WST-1 reduced by superoxide in 8 minutes was determined for each concentration of xanthine oxidase tested. A linear curve was fit to the line (R-squared value = 0.988).
(PDF)

Figure S2 **Treatment with DPI does not affect phagocyte viability.** Human PMN (**A**) and murine peritoneal macrophage (**B**) viability following sustained treatment with 10 µM diphenylene iodinium (DPI). Relative phagocyte viability was determined by microscopy of cells plated in chambered slides as cytosolic exclusion of trypan blue (n = 700 cells scored as 7 objective fields with 100 cells per field). Time points monitored correspond to the times of initial addition of DPI (−20 minutes), time of addition of yeast (0 minutes), and time points post-infection (60 minutes and 240 minutes). Error bars represent standard deviations. No significant differences between DPI-treated and untreated cell viability were found at any time point (p>0.05).
(PDF)

Figure S3 **Lungs infected with Sod3-deficient** ***Histoplasma*** **yeast have reduced inflammation.** Histology of murine lung sections after infection with *Histoplasma* yeast. Wild-type C57BL/6 mice were intranasally infected with approximately 1×10^4^ *SOD3(+)* (OSU45), *sod3Δ* (OSU15) or *sodΔ/SOD3* (OSU49) *Histoplasma* yeasts. At 4 days (**A**) or 8 days (**B**) post-infection, lungs were removed, fixed in 5% formalin, and sections stained with hematoxylin and eosin. Representative images are shown. Scale bars represent 1 mm.
(PDF)

Figure S4 **Intracellular Sod1 function is dispensable for** ***Histoplasma*** **virulence.** Kinetics of sublethal lung infection with *Histoplasma* yeasts. Wild-type C57BL/6 mice were intranasally infected with approximately 2×10^4^ *gfp*-RNAi (OSU104) or *gfp:SOD1*-RNAi (OSU105) *Histoplasma* yeasts. The fungal burden in lungs was determined by quantitative platings for *Histoplasma* cfu at the indicated times representing progressing infection. Each data point represents cfu counts per lung from an individual animal (n = 4 per time point) and horizontal bars represent the mean fungal burden. No significant differences (ns) in fungal burden are detected between infections with the SOD1-proficient strain (*gfp*-RNAi) and the SOD1-depleted strain (*gfp:SOD1*-RNAi). The actual inoculum dose is shown in graphs at day 0. The limit of detection is 100 cfu.
(PDF)
