# Supplementary figures and images for: Extracellular Superoxide Dismutase Protects Histoplasma Yeast Cells from Host-Derived Oxidative Stress
Source: PLoS Pathog. 2012 May 17;8(5):e1002713. doi: 10.1371/journal.ppat.1002713 (PMC3355102; doi:10.1371/journal.ppat.1002713)

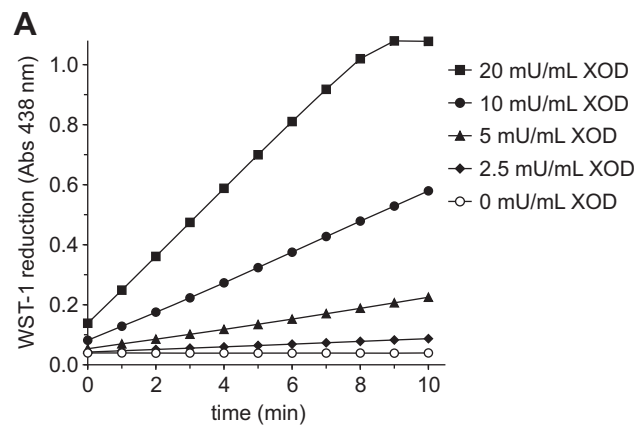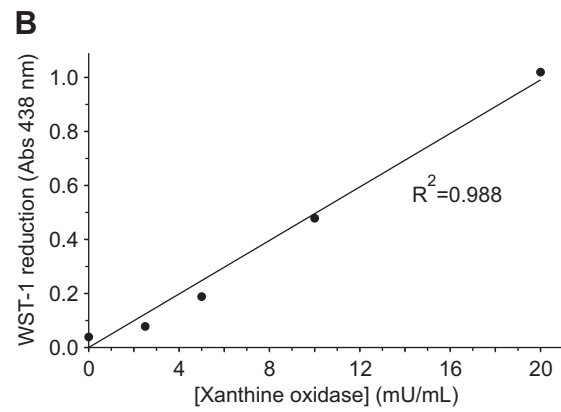

Supplement: Figure S1 — Increasing amounts of xanthine oxidase increase the amount of superoxide produced. (A) Production of superoxide by increasing amounts of xanthine oxidase (XOD) with hypoxanthine. A two-fold dilution series of xanthine oxidase was added to 100 mM hypoxanthine and the superoxide produced was detected by reduction of WST-1. WST-1 reduction was monitored over time by absorbance at 438 nm. Data points represent the mean value of triplicate samples. (B) Linear relationship between the amount of xanthine oxidase and the superoxide produced. The amount of WST-1 reduced by superoxide in 8 minutes was determined for each concentration of xanthine oxidase tested. A linear curve was fit to the line (R-squared value = 0.988). (PDF) [file ppat.1002713.s001.pdf]

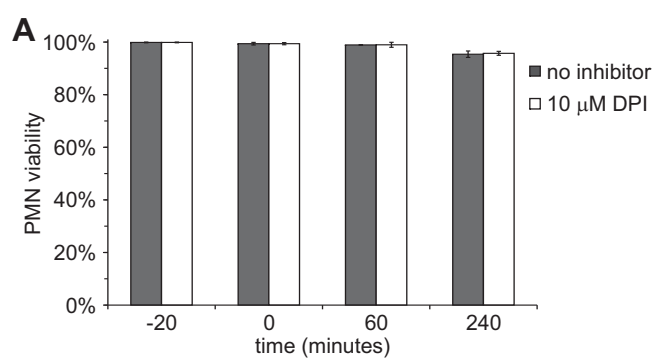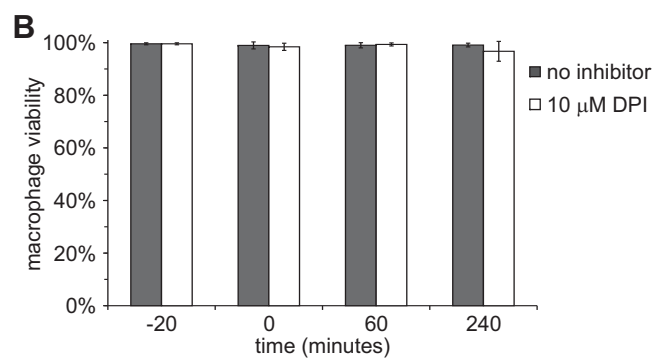

Supplement: Figure S2 — Treatment with DPI does not affect phagocyte viability. Human PMN (A) and murine peritoneal macrophage (B) viability following sustained treatment with 10 µM diphenylene iodinium (DPI). Relative phagocyte viability was determined by microscopy of cells plated in chambered slides as cytosolic exclusion of trypan blue (n = 700 cells scored as 7 objective fields with 100 cells per field). Time points monitored correspond to the times of initial addition of DPI (−20 minutes), time of addition of yeast (0 minutes), and time points post-infection (60 minutes and 240 minutes). Error bars represent standard deviations. No significant differences between DPI-treated and untreated cell viability were found at any time point (p>0.05). (PDF) [file ppat.1002713.s002.pdf]

**A***SOD3(+)**sod3Δ**sod3Δ/SOD3*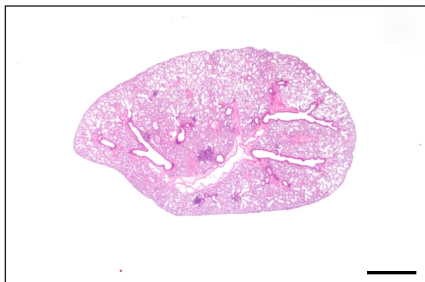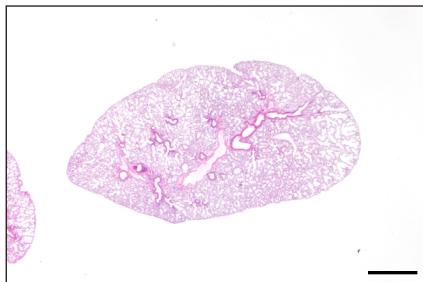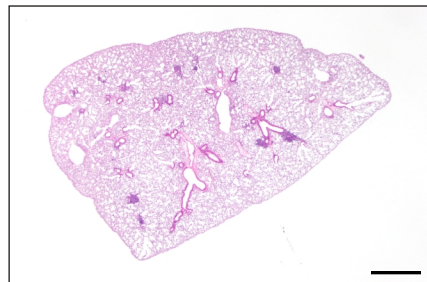**B***SOD3(+)**sod3Δ**sod3Δ/SOD3*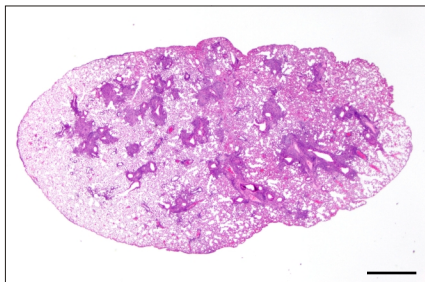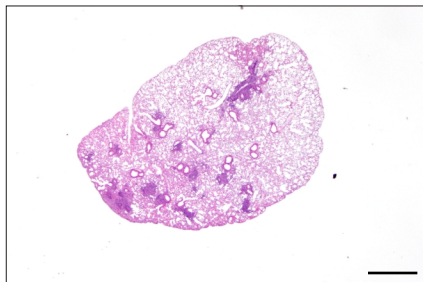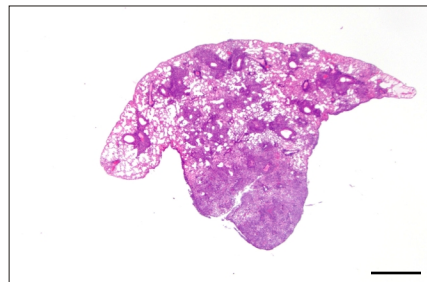

Supplement: Figure S3 — Lungs infected with Sod3-deficient Histoplasma yeast have reduced inflammation. Histology of murine lung sections after infection with Histoplasma yeast. Wild-type C57BL/6 mice were intranasally infected with approximately 1×10^4^ SOD3(+) (OSU45), sod3Δ (OSU15) or sodΔ/SOD3 (OSU49) Histoplasma yeasts. At 4 days (A) or 8 days (B) post-infection, lungs were removed, fixed in 5% formalin, and sections stained with hematoxylin and eosin. Representative images are shown. Scale bars represent 1 mm. (PDF) [file ppat.1002713.s003.pdf]

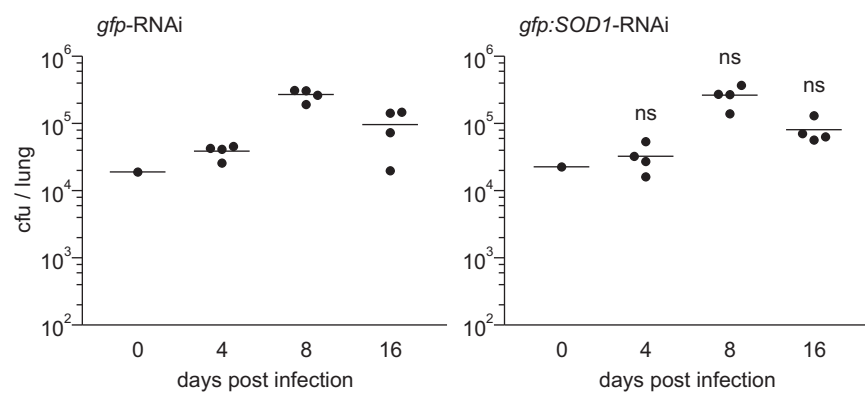

Supplement: Figure S4 — Intracellular Sod1 function is dispensable for Histoplasma virulence. Kinetics of sublethal lung infection with Histoplasma yeasts. Wild-type C57BL/6 mice were intranasally infected with approximately 2×10^4^ gfp-RNAi (OSU104) or gfp:SOD1-RNAi (OSU105) Histoplasma yeasts. The fungal burden in lungs was determined by quantitative platings for Histoplasma cfu at the indicated times representing progressing infection. Each data point represents cfu counts per lung from an individual animal (n = 4 per time point) and horizontal bars represent the mean fungal burden. No significant differences (ns) in fungal burden are detected between infections with the SOD1-proficient strain (gfp-RNAi) and the SOD1-depleted strain (gfp:SOD1-RNAi). The actual inoculum dose is shown in graphs at day 0. The limit of detection is 100 cfu. (PDF) [file ppat.1002713.s004.pdf]
